# Spectrum and epidemiology of neurological disorders and neuromuscular anomalies in pediatric population of Sialkot, Pakistan

**DOI:** 10.12669/pjms.42.5.14103

**Published:** 2026-05

**Authors:** Hamna Shahid, Aneeta Kumari, Urwa Hafeez, Mubara Khizer, Sajid Malik, Sara Mumtaz

**Affiliations:** 1 Hamna Shahid, Human Genetics Section, National University of Medical Sciences, Rawalpindi, Pakistan; 2 Aneeta Kumari, Human Genetics Program, Department of Zoology, Quaid-i-Azam University, Islamabad, Pakistan; 3 Urwa Hafeez, Human Genetics Program, Department of Zoology, Quaid-i-Azam University, Islamabad, Pakistan; 4 Mubara Khizer,Pediatric Department, Allama Iqbal Memorial Teaching Hospital, Sialkot, Pakistan; 5 Sajid Malik, Human Genetics Program, Department of Zoology, Quaid-i-Azam University, Islamabad, Pakistan; 6 Sara Mumtaz, Human Genetics Section, National University of Medical Sciences, Rawalpindi, Pakistan

**Keywords:** Birth defects, Consanguinity, Cerebral palsy, Developmental delay, Genetic defects, Intellectual disability, Pediatric neurology

## Abstract

**Background and Objective:**

Neurological diseases and neuromuscular abnormalities are intricate conditions that impact both the central and peripheral nervous systems. This study aimed to examine the frequency, patterns, phenotypic features, and familial characteristics of pediatric neurological disorders and neuromuscular anomalies in the Sialkot district of Pakistan.

**Methodology:**

A cross-sectional study was performed, with patients recruited from tertiary care hospitals throughout the Sialkot district from February, 2024 to December, 2024. Descriptive statistics were used to analyses and compare the data.

**Results:**

We collected 395 index cases, 65% of which were males. The average age was 10.2±7.2 years. Most of these individuals lived in rural areas (68%) and came from Punjabi-speaking families (96%). Neurological disorders were more prevalent (61%) than neuromuscular anomalies (39%). Intellectual disability (ID) (25%) was the most common neurological disorder, followed by Down syndrome (10%), developmental delay (6.5%), and pediatric seizures (5.5%). Cerebral palsy (CP) was the most common neuromuscular disorder, making up 38.7% of cases. There were also spastic (18.9%), athetoid (14.4%), and ataxic (3.5%) subtypes. There were more sporadic cases (68%) than familial cases (32%). Parental consanguinity was documented in 52% of cases. Most of the time, first-order births were affected (30%).

**Conclusion:**

This study points out a substantial frequency of pediatric neurological disorders and neuromuscular anomalies in the Sialkot district, particularly emphasizing intellectual disability (ID) and cerebral palsy (CP). High levels of consanguinity and familial aggregation indicate a significant genetic influence. These findings highlight the pressing necessity for improved community-based screening, genetic counseling, and enhanced access to multidisciplinary care, especially in rural areas.

## INTRODUCTION

A broad range of complicated medical conditions that interrupt the normal development and subsequent functioning of central and peripheral nervous system are studied under the umbrella of neurological disorders and neuromuscular anomalies.^1^,^2^ Intellectual disability (ID), hypotonia, seizures, delayed developmental milestones and behavioral abnormalities are among the common characteristics of the many diverse and frequently overlapping phenotypes.^3^

It is estimated that more than one in three people worldwide lives with a neurological condition, although prevalence rates vary significantly across regions and age groups.^4^ In 2019 alone, neurological disorders accounted for nearly 10 million deaths and 349 million disability-adjusted life years (DALYs) worldwide.^5^ Notably, over 80% of these deaths and DALYs occurred in low- and middle-income countries (LMICs), where access to diagnosis and treatment remains limited.

ID is a common consequence of many congenital neurological disorders. Globally, approximately 2.4% of children under the age of five, equivalent to 16.1 million children, are affected by ID.^6^ Among congenital causes, Down syndrome remains one of the most prevalent chromosomal etiologies to ID, with an estimated global incidence of 1 in 700 live births.^7^ similarly, cerebral palsy (CP), the most common motor disability in childhood, continues to pose diagnostic and therapeutic challenges.^8^ Pediatric epilepsy is another prevalent neurological disorder, often co-occurring with intellectual disabilities.^9^ In countries like Pakistan, healthcare systems face significant obstacles, including widespread rural populations, inadequate healthcare infrastructure, limited availability of diagnostic tools, and a severe shortage of trained professionals.^10^

Sialkot, Pakistan, is a densely populated district with an urban population now estimated at 3.9 million residents, reflecting rapid demographic growth and associated public health pressures. The city’s industrial base has been linked to environmental pollution that contributes to respiratory, dermatological, hepatic, and neurological disorders, adding to an already high disease burden.^11^ Previously, a community-based study in Sialkot district reported 241 subjects with congenital anomalies, with limb defects (47%) and neurological disorders (31%) being the most frequent categories, highlighting a substantial burden of birth defects.^12^ Despite this high burden, Sialkot suffers from a marked paucity of large scale, population based epidemiological studies on congenital neuro-developmental disorders. Against this scenario, the present study was aimed to investigate the pattern and phenotypic characteristics of neurological disorders and neuromuscular anomalies among the pediatric population of Sialkot district of Pakistan.

## METHODOLOGY

This study was conducted in accordance with the ethical principles outlined in the revised Declaration of Helsinki. Reporting followed the Strengthening the Reporting of Observational Studies in Epidemiology (STROBE) guidelines.^13^

### Ethical Approval:

It was approved by the Institutional Review Board of the National University of Medical Sciences (No.06/IRB&EC/NUMS/40; dated: December 8, 2023).

### Study design and setting:

We conducted a hospital-based, cross-sectional study to identify and characterize pediatric patients presenting with neurological disorders and congenital neuromuscular anomalies. The study was carried out in the pediatric and neonatal departments of two major tertiary care hospitals in Sialkot, Pakistan: Allama Iqbal Memorial Teaching Hospital and Government Sardar Begum Teaching Hospital. Data collection spanned from February 2024 to December 2024.

### Participants and sampling:

The source population consisted of all children admitted to or presenting at the outpatient pediatric and neonatal departments of the participating hospitals during the study period. We employed a consecutive sampling technique to recruit participants. All pediatric patients diagnosed with a neurological disorder or neuromuscular anomaly by the attending physician were considered eligible for inclusion. Patients with incomplete medical records or those whose guardians declined to provide consent were excluded from the study.

### Data collection instrument and procedure:

A structured questionnaire was used to collect data through face-to-face interviews with parents/legal guardians and a review of available medical records. The questionnaire captured a range of biodemographic and socioeconomic variables.

To ensure comprehensive clinical characterization, the following procedures were followed for each index case: phenotypic documentation, clinical classification based on the attending physician’s diagnosis and available clinical data, and family history (familial or sporadic). A three-generation pedigree was constructed for each family to document the pattern of segregation and to identify other affected family members. While pedigrees were drawn for all families, only the data from the index case were included in the final statistical analysis to avoid clustering effects.

### Classification of anomalies and statistical methods:

*S*pecialized clinicians and pediatricians made diagnosis of anomalies. The International Classification of Diseases (ICD-10) and Online Mendelian Inheritance in Man (OMIM) were used for standardized diagnostic definitions and coding systems. The Diagnostic and Statistical Manual of Mental Disorders, Fifth Edition (DSM-5) criteria were used as across-referenced for mental and neurodevelopmental anomalies.^14^

The two primary categories including neurological and neuromuscular disorders were mainly focused. However, the instances with multiple anomalies, the major observation was considered as the primary diagnosis (neurological or neuromuscular). While the additional features were diagnosed as associated (syndromic) manifestations. Infectious and traumatic disorders were excluded from the study.

For the evaluation of variable distributions descriptive statistics were used. Cross-tabulations of categorical variables were performed. The Chi-square or Fisher’s exact tests were used to assess statistical significance. For each anomaly, proportions and 95% confidence interval (CI) were calculated. To explore associations with disease category, bivariate logistic regression was performed for each independent (demographic) variable. All the variables were included in a multivariate logistic regression model. This model was used to determine the independent predictors of disease category after controlling for potential confounders. A p-value of < 0.05 was considered statistically significant, and results are reported as odds ratios (OR) with 95% confidence intervals (CI). Data were analyzed using GraphPad Prism (Ver.5) and STATA (Ver.11).

## RESULTS

### Sample characteristics:

A total of 395 index cases were included in the study, comprising 258 males (65%) and 137 females (35%). Half of the participants were nine years of age or younger with mean age of 10.2±7.2 years. A majority (68%) resided in rural areas, and most belonged to Punjabi-speaking families (96%). In terms of socio-economic status, over half the sample (53%) fell within the lower-middle economic quartile, and the majority (63%) belonged to nuclear families.

### Neurological disorders and neuromuscular anomalies and their socio-demographic predictors:

In this cohort, 242 cases (61%) were classified as having neurological disorders, while 153 (39%) had neuromuscular anomalies. Bivariate and multivariate logistic regression was separately performed for two disease categories, in order to observe the associations with demographic variables.

For neurological disorders, strong protective effect for urban origin (aOR: 0.51), and caste-systems like Gujjar and Malik (aOR: 0.17 and 0.28, respectively) was evident (Table-I). The “Mid” economic status group had significantly lower odds (aOR: 0.54, p <0.05) compared to the “Low-Mid” reference group. Further, “Low” economic status group showed a trend toward higher odds in the crude analysis (OR: 2.30), but this was not significant after adjustment. While extended family was a risk factor in the crude model (OR: 1.69), this association lost significance after adjustment.

**Table-I:** Crude (OR) and adjusted odds ratios (aOR) for neurological disorders and neuromuscular anomalies by sociodemographic characteristics.

Variables	Neurological disorders		Neuromuscular anomalies	
	OR	aOR	OR	aOR
***Gender: male (Ref. female)***	1.00	1.10	1.00	0.91
***Age: >9 (Ref. ≤9 yrs)***	0.79	0.73	1.27	1.36
***Origin: Rural (Ref. urban)***	0.44***	0.51*	2.27***	1.96*
***Caste system: (Ref. Rajput)***				
Jutt	0.77	0.79	1.29	1.26
Malik	0.27**	0.28*	3.73**	3.55*
Mehr	0.53	0.58	1.88	1.71
Gujjar	0.14**	0.17**	7.06**	5.87**
Others	0.57	0.59	1.75	1.70
***Mother tongue: others (Ref. Punjabi)***	1.14	0.84	0.87	1.20
***Economic status: (Ref. low-mid)***				
Poor	0.70	0.68	1.42	1.46
Low	2.30*	1.75	0.44*	0.57
Mid	0.64	0.54*	1.57	1.84*
High	0.94	0.39	1.07	2.59
***Family type: extended (Ref. nuclear)***	1.69*	1.53	0.59*	0.65
***Consanguinity: yes (Ref. no)***	1.08	0.92	0.93	1.09
_cons		4.69***		0.21***

*, p<0.05; **, p<0.01; ***, p<0.001; Ref., reference category.

For neuromuscular disorders, rural origin was a significant risk factor (aOR: 1.96). Individuals from rural areas have nearly twice the odds of having a neuromuscular disorder compared to those from urban areas (Table-I). The Malik caste (vs. Rajput) had significantly higher odds (aOR: 3.55). Gujjar (vs. Rajput) caste showed the strongest risk effect (aOR: 5.87), with nearly six times the odds of neuromuscular disorders compared to the Rajput reference group. The “Mid” economic status group was associated with significantly higher odds (aOR: 1.84) of neuromuscular disorders. Extended family appeared protective in the crude model (OR: 0.59), but this was not significant after adjustment.

The overall regression analysis revealed that sociodemographic factors, particularly caste-system and rural/urban origin, were significant independent predictors of both neurological and neuromuscular disorders, but in opposite directions.

### Pattern of neurological disorders and neuromuscular anomalies:

**Fig.1:**
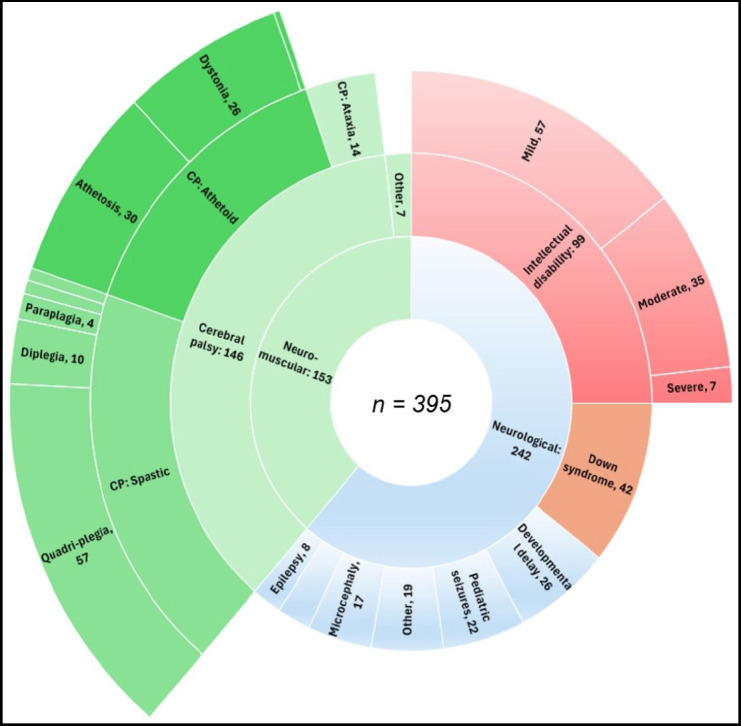
Sunburst chart depicting the distribution and sub-classification of neurological disorders and neuromuscular anomalies.

Neurological disorders encompassed 22 distinct clinical entities, whereas neuromuscular anomalies were grouped into 15 subtypes (Fig.1; Table-II).

**Table-II:** Distribution and relative proportions of major and minor categories of neurological disorders and neuromuscular anomalies.

Major/minor category	No.	Proportion	95% CI	OMIM	ICD-10
***Neurological disorders***	242	0.613	0.565-0.661		
Intellectual disability (ID; all)	99	0.251	0.208-0.293	300243	F79
ID; mild	(57)	0.144	0.110-0.179	249500	F70
ID; moderate	(35)	0.089	0.061-0.117		F71
ID; severe	(7)	0.018	0.005-0.031	611091	F72
Down syndrome	42	0.106	0.076-0.137	190685	Q90
Developmental delay	26	0.066	0.041-0.090	618330	Z13.42
Pediatric seizures	22	0.056	0.033-0.078		P90
Microcephaly	17	0.043	0.023-0.063	251200	Q02
Hydrocephaly	9	0.023	0.008-0.038	236600	G91.9
Epilepsy	8	0.020	0.006-0.034	117100	G40.9
Cong. aganglionic megacolon	4	0.010	0.000-0.020	142623	Q43.1
Meningomyelocele	3	0.008	-0.001-0.016	182940	Q05
Hypothyroidism	2	0.005	-0.002-0.012	275200	E03.1
Niemann-pick disease	2	0.005	-0.002-0.012	257200	E75.2
Cerebral atrophy	1	0.003	-0.002-0.007	618501	G31.9
Distal peripheral neuropathy	1	0.003	-0.002-0.007	162400	G60.9
Dolichocephaly	1	0.003	-0.002-0.007		Q67.2
Dravet syndrome	1	0.003	-0.002-0.007	607208	G40.4
Dropped head syndrome	1	0.003	-0.002-0.007		
Ischemic stroke	1	0.003	-0.002-0.007	601367	I63.9
Macrocephaly	1	0.003	-0.002-0.007	153470	Q75.3
Russell-Silver syndrome	1	0.003	-0.002-0.007	180860	Q87.1
***Neuromuscular anomalies***	153	0.387	0.339-0.435		
Cerebral palsy (CP; all)	146	0.370	0.322-0.417	605388	G80.0
CP: Spastic	(75)	0.190	0.151-0.229		G80
Quadriplegia	(57)	0.144	0.110-0.179	603513	G80.0
Diplegia	(10)	0.025	0.010-0.041		G80.1
Paraplegia	(4)	0.010	0.000-0.020	270800	G11.4
Hemiplegia	(2)	0.005	-0.002-0.012		G80.2
Monoplegia	(2)	0.005	-0.002-0.012		G80.8
CP: Athetoid	(57)	0.144	0.110-0.179		G80.3
Athetosis	(30)	0.076	0.050-0.102		G80.3
Dystonia	(26)	0.066	0.041-0.090		G24
Choreo-athetosis	(1)	0.003	-0.002-0.007		G25.5
CP: Ataxia	(14)	0.035	0.017-0.054	605388	G80.4
Multiple sclerosis	3	0.008	-0.001-0.016		G35
Bardet-Biedl syndrome	2	0.005	-0.002-0.012	209900	Q87.89
Floppy baby syndrome	2	0.005	-0.002-0.012	300868	P94.2

Cong., congenital.

Among the neurological disorders, the most frequently diagnosed condition was ID, present in 99 cases, followed by Down syndrome (42), developmental delay (26), and pediatric seizures (22). Within the ID category: mild ID accounted for 58% of cases; moderate ID for 35%; and severe ID for 7% (Table-II). In the neuromuscular anomalies, CP was more prominent, and observed in 146 of 153 cases (95%). Subtypes of CP included: spastic CP (51%), athetoid CP (39%), and ataxic CP (10%).

### Sporadic/familial nature and disease onset:

Most cases were sporadic (68%), and 32% were familial. Notably, 90% of affected individuals had early-onset disease (Table-III). This was particularly marked in neurological disorders, where 96% had early onset, compared to 83% among neuromuscular anomalies. Conversely, late-onset presentations were more common in neuromuscular anomalies (17% vs. 4%, *p* < 0.0001) (Table-II).

**Table-III:** Family attributes of patients with neurological disorders and neuromuscular anomalies.

Variable	Neurological disorders, n (%)	Neuromuscular anomalies, n (%)	Total, n (%)	P-value
***Familial/sporadic***				
Sporadic	159 (66)	111 (73)	270 (68)	0.154
Familial	83 (34)	42 (27)	125 (32)	
***Disease onset***				
Early	182 (96)	111 (83)	293 (90)	<0.0001
Late	8 (4)	23 (17)	31 (10)	
***Parity***				
1	61 (28)	49 (32)	110 (30)	0.373
2	45 (20)	32 (21)	77 (21)	
3	41 (19)	27 (18)	68 (18)	
4	36 (16)	14 (9)	50 (13)	
>4	38 (17)	29 (19)	67 (18)	
Generations with disease*				
1	41 (49)	25 (60)	66 (52)	0.231
2+	44 (51)	17 (40)	61 (48)	
Sibship with disease*				
1	55 (66)	25 (60)	80 (64)	0.459
2+	28 (34)	17 (40)	45 (36)	
***Total affected family members***				
Males	228 (62)	139 (65)	367 (63)	0.598
Females	137 (38)	76 (35)	213 (37)	
Sum	365 (63)	215 (37)	580 (100)	

* in familial cases

### Parity and pedigree attributes:

Parity analysis revealed that most affected individuals were first parity births (30%), followed by second parity (21%) (Table-III). Pedigree analysis of familial cases showed that 52% of the anomalies were limited to one generation, while 48% spanned two or more generations, suggesting a mix of autosomal recessive and potentially dominant or complex inheritance patterns. In terms of affected siblings, 64% of families had the involvement of one sibship, while 36% had two or more affected sibships (Table-III). Across all families studied, a total of 580 affected individuals were reported, with a clear male predominance (63%). In summary, in the pedigree and parity variables listed, the differences between neurological disorders and neuromuscular anomalies were statistically not significant.

## DISCUSSION

This hospital based study evaluated the pattern, and phenotypic and genetic attributes of neurological disorders and neuromuscular anomalies in a multiethnic population from Sialkot, Pakistan. The present study’s sample was predominantly male (65%), which aligns with the well-documented male preponderance in many neurodevelopmental and neuromuscular conditions, potentially reflecting biological susceptibility or referral patterns.^15^

In this cohort, neurological disorders were more frequent compared to neuromuscular anomalies (≈3:2 ratio). The most frequent neurological disorders were intellectual disability (ID), Down syndrome, and developmental delay. The high frequency of ID (25%) underscores its major contribution to neurodevelopmental morbidity. Within ID, the majority were mild (58%) and moderate (35%) cases, with severe ID comprising only 7%. This distribution is comparable to community-based surveys in India and Pakistan, where mild to moderate ID disproportionately represents cases that come to clinical attention, whereas severe ID may be under-ascertained due to competing mortality or care institutionalization.^16^-^17^ Down syndrome was observed in 42 individuals (10.6%), which is relatively high and may reflect improved clinical recognition and maternal age effects.

Among the neuromuscular anomalies, CP was the most frequent entity in our cohort. The predominance of spastic and athetoid types aligns with global trends, although the relatively higher proportion of athetoid CP may be related to neonatal hyperbilirubinemia or kernicterus, which remain preventable causes in low-resource settings.^6^,^8^,^18^ The high occurrence of CP may also result from perinatal hypoxia, prematurity, or maternal infections.^3^,^19^

Further, age distribution of index cases showed that younger patients had high representation of neurological disorders and whereas neuromuscular anomalies were more pronounced in elder age group. This likely reflects early recognition of severe neurological disorders due to evident developmental delays and seizures, whereas hereditary neuromuscular anomalies, such as muscular dystrophies or certain types of CP, often manifest later as muscle weakness and motor dysfunction become more apparent. These results are in line with both international data and previous studies from Pakistan, which consistently show neurological disorders, particularly ID, as the predominant category among pediatric patients. Similar findings have been reported from Rawalpindi and Hazara region of Pakistan, where neurological conditions outnumbered neuromuscular anomalies.^20^,^21^ This persistent burden in developing settings is attributed to factors such as premature birth, malnutrition, birth asphyxia, perinatal infections, and consanguineous marriages. In Pakistan, many cases of ID are linked to late or missed diagnosis of treatable metabolic disorders.^22^

A striking finding of this study is that sociodemographic factors, particularly urban/rural origin and caste, emerged as significant independent predictors of both neurological disorders and neuromuscular anomalies, but with opposite directions of association. This may suggest distinct etiopathogenic pathways and risk profiles for these two disease categories. The lower odds of neurological disorders in rural areas and certain castes may reflect a combination of higher socioeconomic status, and better access to antenatal care.^5^ This study revealed that neuromuscular anomalies were significantly more frequent among cases originating from rural areas compared to neurological disorders. This disparity may reflect the influence of inadequate perinatal care services, limited access to tertiary care hospitals, diagnostic and rehabilitation facilities, and higher exposure to environmental or nutritional risk factors prevalent in rural communities.^17^

Parental consanguinity, observed in 52% of cases, is consistent with national figures and reinforces its established role in autosomal recessive neurodevelopmental and neuromuscular anomalies.^20^,^23^,^24^ Although consanguinity rates did not differ significantly between the two disorder groups, recessive inheritance likely contributes to both. Familial clustering was found in 32% of cases, with pedigree analysis indicating single-generation (53%) and multigenerational (47%) patterns, suggesting possible autosomal recessive and dominant or X-linked inheritance, respectively.^20^,^24^ These findings underscore the importance of genetic counseling and molecular diagnostics in populations with high rates of inbreeding.

### Strengths of study:

The strengths of study include the detailed documentation of neurological disorders and neuromuscular anomalies and utilization of standard instruments like DSM-5, ICD-10 and OMIM. This multi-system approach ensures comprehensive patient care, accurate diagnosis, and up-to-date genetic understanding, all essential in modern neurology practice.

### Limitations:

First, it does not report true prevalence of anomalies but only reports the pattern and distribution of anomalies. Secondly, it is a cross-sectional study and cannot infer causality or disease progression. Long-term outcomes of early-onset disorders, particularly CP and ID, were not assessed. Further, the predominantly Punjabi-speaking cohort limits generalizability to other ethnic groups. Environmental exposures, obstetric complications, and maternal health factors, important contributors to these conditions, were not investigated.

## CONCLUSIONS

This study demonstrates that neurological disorders and neuromuscular anomalies in this cohort are associated with distinct and opposing sociodemographic risk profiles. Rural origin and specific caste groups (Gujjar, Malik) were protective against neurological disorders but conferred significantly higher odds of neuromuscular anomalies, highlighting divergent etiological pathways. The clinical burden was dominated by ID among neurological disorders and CP, with a notable excess of athetoid subtypes, among neuromuscular anomalies. The predominantly sporadic (68%) and early-onset (90%) nature of these conditions underscores the critical importance of early detection and intervention. These findings call for targeted public health strategies that address rural-urban disparities, improve perinatal and neonatal care in high-risk populations, and incorporate genetic counseling in communities with strong caste-based clustering of disease.

### Authors’ Contribution:

**SMk and SMz:** conceptualization and supervision.

**HS, AK, UH, MK:** data acquisition and analyses.

**HS and SMk:** statistical analysis and manuscript writing.

All authors gave approval of final version.

**SMz** is accountable for all aspects of the work.
